# Exploring Additional Valuable Information From Single-Cell RNA-Seq Data

**DOI:** 10.3389/fcell.2020.593007

**Published:** 2020-12-01

**Authors:** Yunjin Li, Qiyue Xu, Duojiao Wu, Geng Chen

**Affiliations:** ^1^Center for Bioinformatics and Computational Biology, Shanghai Key Laboratory of Regulatory Biology, Institute of Biomedical Sciences, School of Life Sciences, East China Normal University, Shanghai, China; ^2^Institute of Clinical Science, Zhongshan Hospital, Fudan University, Shanghai, China

**Keywords:** single-cell RNA-seq, cell-to-cell communication, RNA velocity, copy number variations, non-coding RNAs, cell-type deconvolution

## Abstract

Single-cell RNA-seq (scRNA-seq) technologies are broadly applied to dissect the cellular heterogeneity and expression dynamics, providing unprecedented insights into single-cell biology. Most of the scRNA-seq studies mainly focused on the dissection of cell types/states, developmental trajectory, gene regulatory network, and alternative splicing. However, besides these routine analyses, many other valuable scRNA-seq investigations can be conducted. Here, we first review cell-to-cell communication exploration, RNA velocity inference, identification of large-scale copy number variations and single nucleotide changes, and chromatin accessibility prediction based on single-cell transcriptomics data. Next, we discuss the identification of novel genes/transcripts through transcriptome reconstruction approaches, as well as the profiling of long non-coding RNAs and circular RNAs. Additionally, we survey the integration of single-cell and bulk RNA-seq datasets for deconvoluting the cell composition of large-scale bulk samples and linking single-cell signatures to patient outcomes. These additional analyses could largely facilitate corresponding basic science and clinical applications.

## Introduction

In recent years, single-cell RNA-seq (scRNA-seq) technologies and related bioinformatics methods have been developing and innovating rapidly, which significantly revolutionized our understanding of the expression heterogeneity and transcriptome dynamics of individual cells for diverse species including human (Quadrato et al., 2017), mouse (Brown et al., 2016), zebrafish (Wagner et al., 2018), and Drosophila (Karaiskos et al., 2017). The data generated by scRNA-seq can be generally grouped into read-based and unique molecular identifier (UMI)-based, depending on the full-length transcript sequencing [e.g., Smart-seq2 (Picelli et al., 2014)] or 3′/5′-end capturing [such as 10× Chromium (Zheng et al., 2017), and Drop-seq (Macosko et al., 2015)] protocols used (Chen et al., 2019). A series of preprocessing steps are required for overcoming the high noise of raw scRNA-seq data to obtain robust results from downstream analysis. Quality control (QC) of scRNA-seq data is important to remove the low-quality cells resulting from RNA degradation, break of the cell membrane, or multicells to avoid misinterpretation of downstream results, which have been reviewed recently (Luecken and Theis, 2019). Then normalization is needed to eliminate the influence of technical effects on molecular counts (e.g., sequencing depth) to make gene expression comparable between cells. For the two main types of data generated from the full-length transcript and 3′/5′-end enrichment scRNA-seq protocols, distinct normalization methods are needed. It is recommended to take gene length into account for full-length transcript scRNA-seq data (such as the common approach of TPM normalization), while disparate methods like scran (Lun et al., 2016) are required for normalizing 3′/5′-tag scRNA-seq data (Luecken and Theis, 2019). However, normalization cannot directly address the biases of technical noises (e.g., batch effect and dropout) and biological covariates (such as cell cycle); further data processing like batch effect correction and imputation may be needed to mitigate such effects according to the data properties and research goals.

After data preprocessing, a range of common analyses can be conducted, like cell type/state identification and annotation, trajectory inference, alternative splicing detection, gene regulatory network (GRN) reconstruction, which has been reviewed by us and other colleagues (Chen et al., 2019; Luecken and Theis, 2019). Because scRNA-seq data usually involve many cells and thousands of genes, feature selection and dimensionality reduction methods are needed to reduce the dimensionality of high-dimensional datasets to lighten the computational burden of downstream analysis (Andrews and Hemberg, 2018). Generally, 500–5,000 highly variable genes are often used depending on the data complexity in feature selection approaches (Yip et al., 2019). Linear [e.g., principal component analysis (PCA)] or non-linear {such as t-distributed stochastic neighbor embedding [t-SNE (van der Maaten and Hinton, 2008)]} and uniform approximation and projection (UMAP) (Diaz-Papkovich et al., 2019) dimensionality reduction methods can be used to further reduce the data dimension and visualize the data in two or three dimensions (Moon et al., 2018).

Based on the data with reduced dimensions, the cell clusters are typically identified in single-cell analysis. Methods for clustering (such as *k*-means) or community detection (e.g., *K*-nearest neighbor graph) are often applied to determine the clusters according to the expression similarity of genes (Duò et al., 2018; Kiselev et al., 2019). Once the clusters of single cells are determined, marker genes can be identified through differential expression (DE) analysis to annotate the clusters with meaningful biological insight. Moreover, for the scRNA-seq data generated from full-length transcript sequencing protocols, the alternative splicing changes between distinct cell clusters can be further investigated as we summarized previously (Chen et al., 2019). On the other hand, for the single-cell datasets involving developmental or differentiation process, trajectory inference methods can be utilized to infer the order of cells along developmental trajectories. Saelens et al. (2019) benchmarked dozens of trajectory inference tools and revealed that these methods are complementary with variable performances depending on the dataset dimensions and trajectory topology. Additionally, cellular differentiation and cell state transition processes are controlled by the underlying GRNs. An increasing number of approaches have been developed to infer the GRNs from scRNA-seq data generally based on the assumption that the genes with similar expression profiles could be regulated by a common transcription factor [such as SCENIC (Aibar et al., 2017)], but more efforts are needed to improve the accuracy of these analytical approaches (Chen and Mar, 2018; Fiers et al., 2018; Pratapa et al., 2020).

**FIGURE 1:**
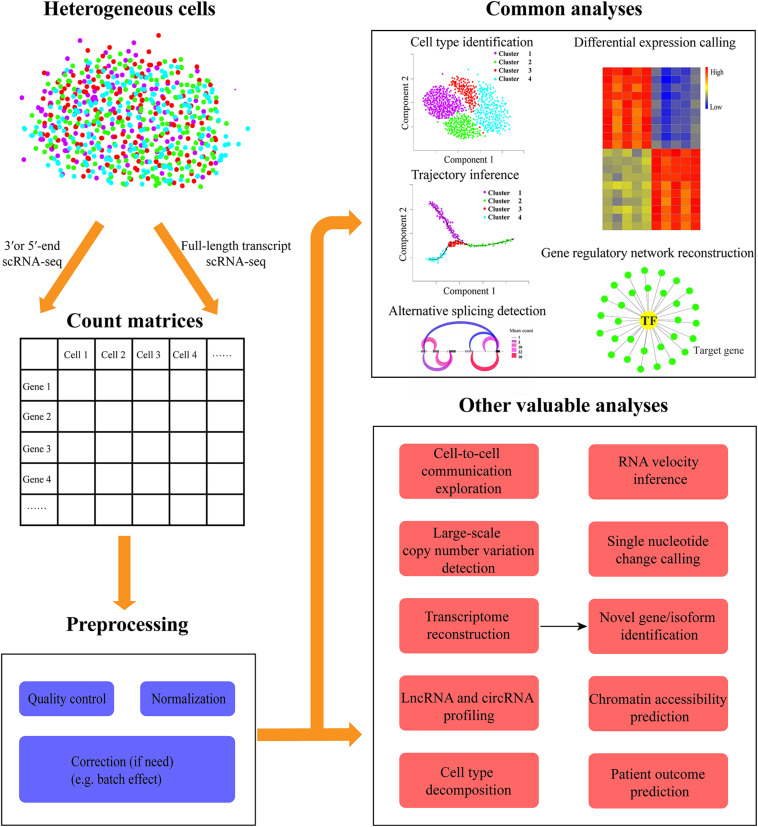
Overview of diverse common and additional valuable analyses of scRNA-seq data. The heterogeneous cells can be sequenced with the full-length transcript or 3′/5′-end capturing scRNA-seq protocols. Then the expression count matrices for all genes in each cell can be quantified from scRNA-seq data. Before downstream analysis, a series of preprocessing steps are needed to be conducted including quality control (e.g., elimination of low-quality cells), normalization, and correction (if need, such as batch effect). The common scRNA-seq data analyses in most studies include cell type identification, differential expression calling, trajectory inference, gene regulatory network reconstruction, and alternative splicing detection. Besides these routine explorations, other valuable analyses can be carried out, such as cell-to-cell communication exploration, RNA velocity inference, large-scale copy number variation, and single nucleotide change detection, chromatin accessibility prediction, transcriptome reconstruction for novel gene/isoform identification, lncRNA, and circRNA profiling, cell type decomposition, and patient outcome prediction.

However, besides those common analyses, many other valuable explorations can be conducted to gain additional insights into scRNA-seq data (Figure 1). In this review, we first describe the progress and related methods for cell–cell communication network inference, RNA velocity analysis, interrogation of chromosomal-scale copy number variations (CNVs) and single nucleotide variations, as well as novel gene/isoform identification. Then we summarize the integration of single-cell and bulk RNA-seq data to cost-effectively analyze a large sample size. In particular, we discuss their implications and potential challenges as well as future directions.

## Cell-To-Cell Communication Network Inference

Cells often do not function independently but can communicate with each other and change their behaviors by transmitting and receiving signals within their environment. In multicellular organisms, cell signaling is critical for joining different cell types together to form tissues (e.g., brain, lung, muscle, and liver). Specifically, autocrine (interact with the same or similar cells) and paracrine (communicate with nearby cells) signaling networks within and across cell types play fundamental roles for cells working together to coordinate diverse organismal processes. Moreover, an abundance of cell fate decisions are made to react to extracellular signals from the interactions between secreted ligands and cell-surface receptors in the local environment (Watabe and Miyazono, 2009). Especially for cancers, the tumor microenvironment is typically composed of various cell types (including malignant, immune, and stromal cells). Understanding the cell-to-cell communication/interaction network among distinct cell populations can facilitate the elucidation of underlying mechanisms for tumorigenesis, tumor progression, metastasis, therapy resistance, and immune infiltration (Hanahan and Weinberg, 2011). Defects in cell-to-cell interaction have been demonstrated to be associated with different cancers (Haass et al., 2004), autoimmune (Gorelik and Flavell, 2000), and metabolic diseases (Hotamisligil, 2006).

**FIGURE 2:**
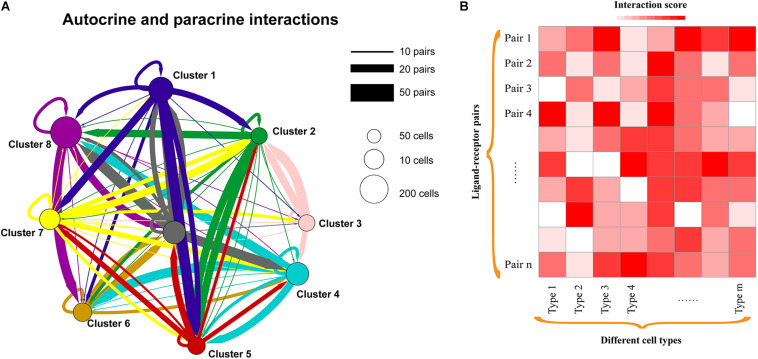
Cell-to-cell communication exploration of different cell types. **(A)** Network view of autocrine and paracrine cell-to-cell communications within and across cell types. Autocrine signaling represents the signaling cells and the target cells that are the same or similar cells (such as belonging to the same cell type), while paracrine signaling could be the interactions between different cell types in a microenvironment. The circles and edges are in proportion to the counts of ligand–receptor interaction pairs. **(B)** Heatmap showing the interaction scores of ligand–receptor pairs in each cell type. Interaction scores could be the significance (e.g., *P*-value) or the weighted scores for ligand–receptor interactions.

ScRNA-seq enables expression quantification of transcripts encoding ligands and their cognate receptors in each cell, which provides unprecedented opportunities for decoding the diversity, complexity, and dynamics of intercellular communication networks (Figure 2). An increasing number of studies investigated the cell-to-cell communications between distinct cell populations and uncovered meaningful biological insights. For example, interlineage communications mediated by ligand–receptor complexes among single cells can regulate liver bud development (Camp et al., 2017), and functionally important ligand-receptor interactions associated with cancer metastasis were recently identified in head and neck squamous cell carcinoma (Puram et al., 2017). We also detected a set of intercellular communications between macrophages and cancer stem-like cells (CSCs) in glioma that the expression levels of involved ligands and receptors are significantly correlated with the survival of patients (Yuan et al., 2019). Moreover, lung basophils were found to widely communicate with both immune and non-immune compartments (Cohen et al., 2018), and cell–cell interactions were useful in identifying the cell types of human placenta (Pavlicev et al., 2017). Interaction network analysis between distinct cell types within the melanoma microenvironment highlighted that tumor cell composition is critical for diagnostic and therapeutic strategies (Tirosh et al., 2016a). Additionally, extensive intercellular communication networks were observed between diverse mouse heart cell types, which contributed to the transcriptional program of sexual dimorphism (Skelly et al., 2018).

To identify the potential interactions within or between cell subpopulations from scRNA-seq data, an increasing number of computational methods have been developed based on the expression abundance of ligand and receptor pairs (Table 1). For instance, Kumar et al. (2018) proposed a computational approach to characterize cell–cell communications across the cell types in a microenvironment (such as tumor ecosystem) by scoring the ligand–receptor interactions between two cell types as the product of average expression of ligands and receptors in corresponding cell types. PyMINEr integrates ligand and receptor information, protein–protein interactions as well as pathway analyses to build the autocrine–paracrine signaling networks (Tyler et al., 2019). scTensor defines the cell–cell interactions as directed hypergraphs (nodes are cell types, and edges represent ligand–receptor pairs) and can infer many-to-many interactions with tensor decomposition (Tsuyuzaki et al., 2019). iTALK identifies the intercellular crosstalk signals based on curated ligand–receptor pairs and can visualize the results in different plot formats like Circos, network, and errorbar (Wang Y. et al., 2019). Moreover, CellPhoneDB provides a repository of curated receptors, ligands, and their interactions, and can allow users to search particular ligand/receptor or predict enriched cellular interactions with inquired scRNA-seq data efficiently (Efremova et al., 2020). CellChat quantitatively infers intercellular communication networks using mass action models, which also enables the visualization of cellular interactions (Jin et al., 2020a). Additionally, SingleCellSignalR allows the assessment of the confidence of predicted ligand–receptor (Cabello-Aguilar et al., 2020), while NicheNet can enable a functional understanding of cell–cell communications by providing the information on how ligand–receptor interactions influence the target gene expression (Browaeys et al., 2020). However, the study for systematic performance evaluation of these methods is currently lacking. Moreover, the available approaches for inferring cell–cell interactions are generally based on the known and/or curated ligand–receptor pairs; the interactions mediated by unknown ligand–receptor pairs will be missed. When interpreting the resulting cell–cell communications between cell types, especially the number of interactions, it would be better to consider the missing interactions. Therefore, dissecting the cellular communications in health and disease states will largely benefit the elucidation of the underlying molecular mechanisms.

**TABLE 1:** Computational approaches for additional analyses of scRNA-seq data.

Categories	Suitable scRNA-seq protocols	Tools	URL	References
Cell to cell communication	Full-length transcript or 3′/5′-tag sequencing	PyMINEr	https://bitbucket.org/scottyler892/pyminer_release	Tyler et al., 2019
		scTensor	https://rdrr.io/bioc/scTensor/	Tsuyuzaki et al., 2019
		iTALK	https://github.com/Coolgenome/iTALK	Wang Y. et al., 2019
		CellPhoneDB	https://github.com/Teichlab/cellphonedb	Efremova et al., 2020
RNA velocity	Full-length transcript or 3′/5′-tag sequencing	velocyto	https://github.com/velocyto-team/velocyto.R	La Manno et al., 2018
		scVelo	https://github.com/theislab/scvelo	Bergen et al., 2019
Copy number variation	Full-length transcript or 3′/5′-tag sequencing	inferCNV	https://github.com/broadinstitute/inferCNV	Patel et al., 2014
		HoneyBADGER	https://github.com/JEFworks/HoneyBADGER	Fan et al., 2018
Chromatin accessibility	Full-length transcript sequencing or 3′/5′-tag sequencing	BIRD	https://github.com/WeiqiangZhou/BIRD	Zhou et al., 2017
Single nucleotide variants	Full-length transcript sequencing	SAMtools	http://samtools.sourceforge.net/	Li, 2011
		Strelka2	https://github.com/Illumina/strelka	Kim et al., 2018
		FreeBayes	https://github.com/ekg/freebayes	Garrison and Marth, 2012
RNA editing	Full-length transcript sequencing	GIREMI	https://github.com/zhqingit/giremi	Zhang and Xiao, 2015
		REDItools	https://github.com/BioinfoUNIBA/REDItools	Picardi and Pesole, 2013
Transcriptome reconstruction	Full-length transcript sequencing	TransComb (genome-guided)	https://zenodo.org/record/61994#.XiEfaOgzaUk	Liu J. T. et al., 2016
		StringTie (genome-guided and *de novo*)	https://ccb.jhu.edu/software/stringtie/	Pertea et al., 2015
		Cufflinks (genome-guided)	http://cole-trapnell-lab.github.io/cufflinks/	Trapnell et al., 2010
		Trinity (*de novo*)	https://github.com/trinityrnaseq/trinityrnaseq/wiki	Grabherr et al., 2011
		*Trans*-ABySS (*de novo*)	https://github.com/bcgsc/transabyss	Robertson et al., 2010
		rnaSPAdes (*de novo*)	http://cab.spbu.ru/software/rnaspades/	Bushmanova et al., 2019
Coding potential assessment	Full-length transcript sequencing	CPAT	http://rna-cpat.sourceforge.net/	Wang et al., 2013
		LncRNA-ID	https://github.com/zhangy72/LncRNA-ID	Achawanantakun et al., 2015
		LGC	http://bigd.big.ac.cn/biocode/tools/BT000004	Wang G. Y. et al., 2019
Circular RNA identification	Total RNA (poly (A+) and poly (A−) RNAs) sequencing	find_circ2	https://github.com/rajewsky-lab/find_circ2	Memczak et al., 2013
		CircExplorer2	https://circexplorer2.readthedocs.io/en/latest/	Zhang et al., 2016
		CIRI2	https://sourceforge.net/projects/ciri/	Gao et al., 2018
Cell composition deconvolution	Full-length transcript or 3′/5′-tag sequencing	CMP	https://cran.r-project.org/web/packages/scBio/index.html	Frishberg et al., 2019
		MuSiC	https://github.com/xuranw/MuSiC	Wang X. R. et al., 2019
		DWLS	https://bitbucket.org/yuanlab/dwls	Tsoucas et al., 2019
		CIBERSORTx	https://cibersortx.stanford.edu/	Newman et al., 2019
Survival analysis	Full-length transcript or 3′/5′-tag sequencing	Cox regression	https://github.com/therneau/survival	Li, 2003

## Reconstruction of Spatial Cellular Communications and Gene Expression

Additionally, the spatial organization of cells is closely associated with diverse cell functions and behaviors including cell–cell interactions, but such information is usually missing from scRNA-seq data as the cells are needed to be dissociated before sequencing. Interestingly, novoSpaRc was recently developed to enable *de novo* spatial reconstruction of gene expression using scRNA-seq data alone (Nitzan et al., 2019). Specifically, CSOmap cannot only predict the cellular interactions but also can infer the cell spatial organizations *de novo* from single-cell transcriptomic data (Ren et al., 2020). Furthermore, the sequencing-based or image-based spatial technologies that can preserve the spatial coordinates of cells have achieved great progress (Mayr et al., 2019). Integrative analysis of the spatial and scRNA-seq data may enable us to gain novel insights into cell–cell communications by constructing the spatial expression patterns of signaling ligands and receptors using transfer learning or deep learning approaches (Efremova and Teichmann, 2020). For instance, SpaOTsc can allow the inference of spatial gene expression patterns and spatial cell–cell communications by incorporating scRNA-seq and spatial data (Cang and Nie, 2020). With the innovation of scRNA-seq and spatial transcriptomics as well as the computational algorithms, the accuracy of intercellular communication network inference will be improved as well. Specifically, such analysis may shed light on the signaling mechanisms of cellular behaviors and responses under various conditions like tumor progression, development, or differentiation.

## Identification of Large-Scale Copy Number Variations

Besides cellular communication detection, scRNA-seq data can be used to identify different types of genomic variations. Intratumoral heterogeneity is a ubiquitous feature for various cancer types, which contributes to tumor progression and therapy failure (Kreso and Dick, 2014). One of the well-studied sources of intratumoral heterogeneity is genetic variation, such as single nucleotide variations and CNVs that are the gains or losses of genomic sequences larger than one kilobase in size (Vogelstein et al., 2013). CNVs play an essential role in generating both physiological and pathological phenotypes through altering corresponding gene transcription or disrupting neighboring or distant non-coding regulatory regions; some of them could have pathogenic roles in common and rare cancers (Shlien and Malkin, 2009).

**FIGURE 3:**
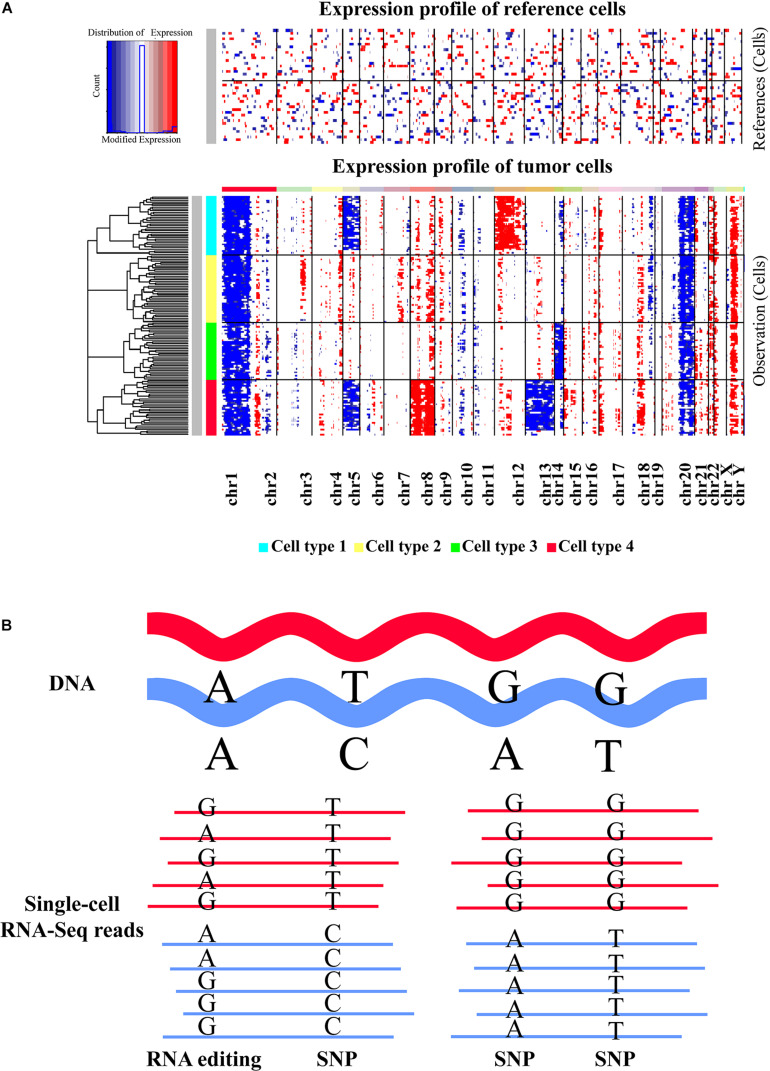
Inference of large-scale copy number alterations and single nucleotide changes based on scRNA-seq data. **(A)** Representative heatmap displaying the large-scale copy number variations (CNVs) identified in different cell types with scRNA-seq data. Top panel shows that no significant large-scale CNVs were identified in reference normal cells, whereas chromosomal-scale deletions (blue) and gains (red) were observed for several chromosomes in different cell subtypes of tumor cells (second panel). The heatmap was created by inferCNV. **(B)** Graphic view of single nucleotide variations and RNA editing events. The reads of scRNA-seq data generated from full-length transcript sequencing protocols are mapped to the reference genome first. Then specific SNV calling tools or RNA-editing detection approaches can be applied to determine the SNVs or RNA-editing events based on the alignment result. Both SNV and RNA editing identifications are mainly suitable for the scRNA-seq methods that can generate full-length transcripts. Moreover, sequencing depth could be an important factor influencing the detection accuracy.

As large-scale CNVs may cause the gain or loss of many genes, they can result in the upregulation or downregulation of the genes in the affected regions. It has been shown that scRNA-seq data can provide informative large-scale CNV evidence for corresponding cells (Figure 3A). For instance, Patel et al. (2014) revealed coherent chromosomal-scale CNV pattern in glioblastoma by averaging relative expression levels of genes over large chromosomal regions and comparing with a set of reference normal cells using their method of inferCNV. With a similar approach, somatic large-scale CNVs were examined in metastatic melanoma (Tirosh et al., 2016a), oligodendroglioma (Tirosh et al., 2016b), as well as head and neck cancer (Puram et al., 2017) at single-cell resolution, which allowed researchers to effectively distinguish malignant cells from non-malignant ones. Recently, another computational method that integrated the hidden Markov model with a Bayesian approach, called HoneyBADGER, has also been developed for identifying the CNVs and loss of heterozygosity in single cells based on the allele and expression information inferred from scRNA-seq data (Fan et al., 2018) (Table 1). Since genomic instability is a hallmark of diverse cancers (Negrini et al., 2010; Ferguson et al., 2015), detecting the somatic large-scale CNVs in single cells could discriminate tumor cells from normal ones and gain insights into their roles in tumorigenesis. However, attention should be paid to the sparsity and noise of scRNA-seq data because currently available scRNA-seq approaches are generally with high-dropout property, which may result in false positives and influence the CNV detection. Collectively, scRNA-seq provides an alternative and cost-effective way for exploring large-scale CNVs in individual cells. It is valuable for unraveling the evolutionary complexity of tumors and understanding cancer development and progression.

## Analysis of Single Nucleotide Variants and RNA Editing

In addition to CNV detection, single nucleotide variants (SNVs) and RNA editing events could also be inferred from single-cell transcriptomic data. SNVs are the most prevalent type of genetic variation and are closely associated with diverse normal and disease phenotypes. The influences of SNVs could manifest on gene expression by *cis* and/or *trans* effects (Bryois et al., 2014), and a multitude of SNVs have been linked to tumor evolution (Navin et al., 2011). Importantly, SNVs in progenitors could be inherited by all the daughter cells during DNA replication, thus systematic SNV calling in single cells is one promising strategy for delineating cellular heterogeneity and phylogenetic relationships, especially for cancer evolution (Navin et al., 2010; Abbosh et al., 2017; Ju et al., 2017; Martincorena et al., 2017). Although single-cell exome sequencing or whole-genome sequencing technologies can be used to interrogate SNVs, such approaches could introduce substantial error rates due to inherent technical limitations (Xu et al., 2012; Zafar et al., 2016), and they are highly expensive for sequencing a large number of cells. By contrast, scRNA-seq is more affordable, and the SNVs detected from single-cell transcriptomic data could be interesting since they are expressed, and their functions are easier to elucidate. A range of studies have revealed intriguing findings by exploring SNVs from scRNA-seq data using the tools originally developed for bulk sequencing data (Tirosh et al., 2016b; Enge et al., 2017; Fan et al., 2018; Poirion et al., 2018; Ding et al., 2019). For example, Enge et al. (2017) gained insights into aging-related genetic and transcriptional processes of the human pancreas by analyzing the somatic mutation patterns with single-cell transcriptomic data. A linear modeling framework, SSrGE, was recently proposed to detect the effective and expressed SNVs that are associated with gene expression from scRNA-seq data, which could facilitate the subpopulation identification and genotype–phenotype relationship determination (Poirion et al., 2018). Moreover, Ding et al. (2019) developed a method for trajectory inference based on the SNPs inferred from scRNA-seq data.

Currently, few tools were specially designed for SNV calling based on single-cell transcriptomic data. However, Liu et al. (2019) systematically evaluated the performance of traditional variant callers on scRNA-seq datasets and recommended SAMtools (Li, 2011), Strelka2 (Kim et al., 2018), and FreeBayes (Garrison and Marth, 2012) to call SNVs for the data with low supporting reads, with sufficient read depths, and with high variant allele frequencies, respectively (Table 1). SAMtools calls the SNVs directly based on the sequencing data with a statistic model, while Strelka2 employs a mixture model to alleviate the effects of context-specific variation, and FreeBayes uses a Bayesian statistical framework to model multiallelic loci. With these tools, the SNVs in each cell can be predicted by treating each cell as a sample like bulk data. Notably, low read depths that resulted from the biologically low expressions and/or technical bias (e.g., dropout events) could reduce the sensitivity of SNV detection. Therefore, the innovation of scRNA-seq strategies to minimize the dropout events will greatly improve the accuracy of SNV inference (Liu et al., 2019). Moreover, novel SNV calling methods that are specifically designed for scRNA-seq are also crucial for correcting the technical bias and increase the sensitivity and specificity of variant calling. Overall, detecting SNVs from single-cell transcriptomic data could provide another layer of cellular heterogeneity among single cells besides gene expression (Figure 3B), which could be useful for lineage tracing and subpopulation identification as well as genotype–phenotype linkage inference (Poirion et al., 2018; Tang, 2020).

Unlike genomic SNVs, RNA editing is a posttranscriptional process that made nucleotide changes on RNA sequences, and adenosine-to-inosine (A-to-I) editing is the most common type in general (Nishikura, 2010) (Figure 3B). RNA editing has been considered as a crucial mechanism for increasing the molecular diversity and regulating the function of proteins (Maas et al., 2006; Park et al., 2012). The known functional impacts of RNA editing mainly include amino acid sequence changes, alternative splicing alteration, RNA stability influence, and alternations on miRNA sequence or miRNA targeting sequence (Nishikura, 2016). Furthermore, aberrant RNA editing events could be correlated with the etiology or progression of various diseases, such as amyotrophic lateral sclerosis, astrocytoma, hepatocellular carcinoma, and metastatic melanoma (Slotkin and Nishikura, 2013; Kung et al., 2018; Kanata et al., 2019). Although sequencing the genome and transcriptome from the same sample/cell can theoretically enable more accurate RNA editing detection, such data are relatively uncommon and costly. Several computational tools are available for robustly identifying RNA editing sites using bulk RNA-Seq data alone, such as GIREMI (Zhang and Xiao, 2015), the pipeline proposed by Ramaswami et al. (2013), and REDItools (Picardi and Pesole, 2013) (Table 1). However, the approaches specifically developed for scRNA-seq data are currently lacking, and a few studies investigated the RNA editome in individual cells. Recently, Ding et al. (2019) suggested that an abundance of SNVs identified from scRNA-seq data by their method are likely to be RNA-editing events. Since aberrant RNA editing events could be correlated with the etiology or progression of many diseases including cancers (Slotkin and Nishikura, 2013; Kung et al., 2018; Kanata et al., 2019), exploring the RNA editome in single cells can facilitate a better understanding of their functional implications to cellular heterogeneity and clinical utility in diseases. Considering that RNA editing detection depends closely on the sequencing depth, applying the tools originally designed for bulk data to single-cell data should be careful due to the inherent technical noise and low sequencing depth of current scRNA-seq protocols. There is an urgent need to develop robust methods for identifying RNA editing events with single-cell data. Consequently, exploring the RNA editome in single cells will be more feasible with the improvement of single-cell sequencing and specialized algorithms, which will benefit the elucidation of the functional implications of RNA editing to cellular variations and disease development.

## Exploring RNA Velocity

**FIGURE 4:**
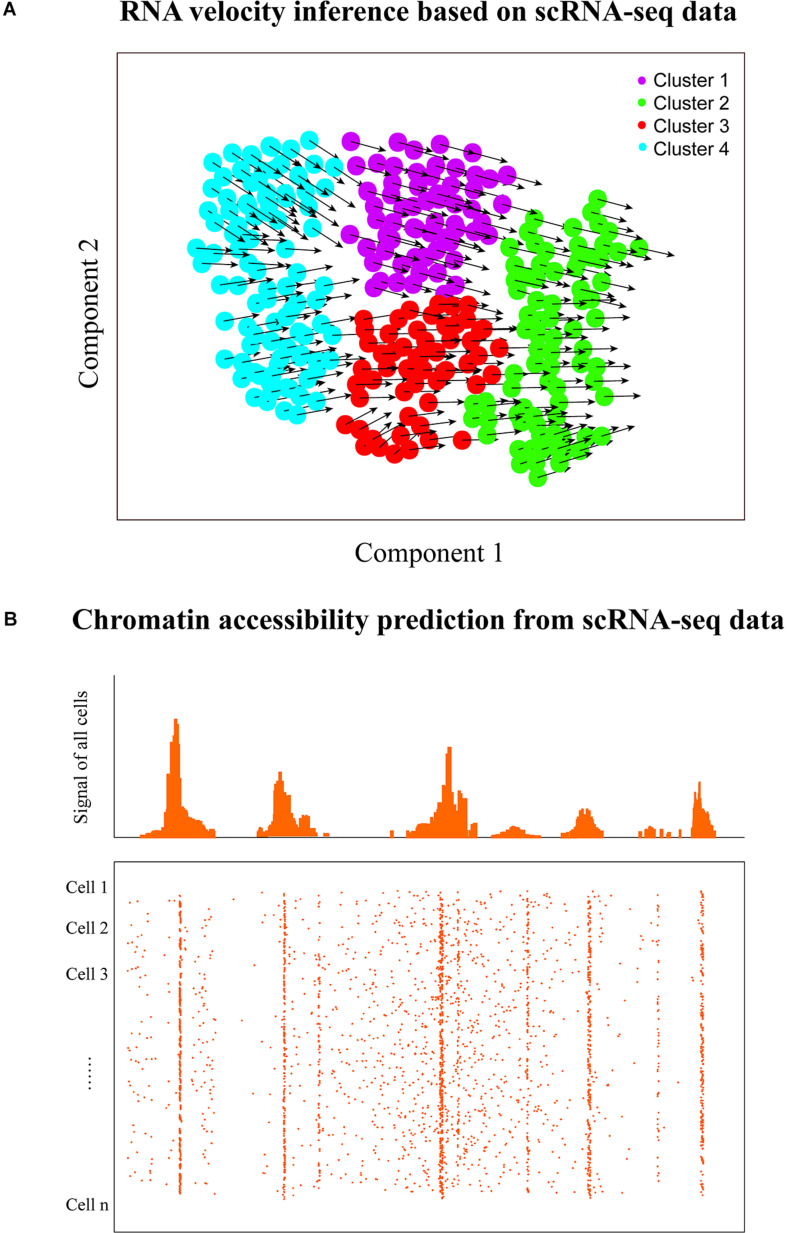
RNA velocity and chromatin accessibility analyses. **(A)** RNA velocity inference of single cells to predict their future transcriptional states. The velocity of gene expression could be represented as the mRNA abundance over time, which enables the prediction of future transcriptional state of cells (the arrows denote the directionality). **(B)** Graphic view of chromatin accessibility prediction with scRNA-seq data. Transcriptome and regulome could have bidirectional interplay because of the feedback, thus scRNA-seq has the potential to predict the chromatin accessibility of transcribed regions using the corresponding computational approach. However, it is worth noting that the chromatin accessibility of non-transcribed regions cannot be predicted with scRNA-seq.

ScRNA-seq data have also been used to predict the future transcriptional state of single cells (termed RNA velocity) by deducing their directed dynamic transcriptome changes (Figure 4A). RNA regulation involves multiple stages including transcription, RNA maturation, and RNA degradation; thus, the abundance of RNAs is a strong indicator of cell state. Previous bulk RNA-seq study has shown that gene splicing and degradation can be effectively estimated based on the relative abundance of unspliced and spliced RNAs (Zeisel et al., 2011; Gaidatzis et al., 2015). Thus, similar signals could be also decoded from individual cells with single-cell transcriptomic data (Svensson and Pachter, 2018). La Manno et al. (2018) proposed a model named velocyto (Table 1) to estimate the rate of change in mRNA abundance (RNA velocity) to predict the future transcriptional state of individual cells by distinguishing between spliced and unspliced mRNAs with scRNA-seq data. This RNA velocity inference method has been applied to an increasing number of researches. For instance, RNA velocity analysis revealed dynamic transcriptional changes of immune cells in hepatocellular carcinoma (Zhang et al., 2019) and could also allow effective identification of the major directions of cell progression for murine neural crest cells (Soldatov et al., 2019). Moreover, Kanton et al. (2019) successfully uncovered the differentiation of neural progenitor cells in human development with RNA velocity exploration, but velocyto could not efficiently process large datasets and even may run out of memory (e.g., cell number >40,000). More recently, Bergen et al. developed a likelihood-based dynamical model, scVelo (can handle >300,000 cells), to infer the RNA velocity of cells by solving the full transcriptional dynamics (Bergen et al., 2019), which is 10 times faster and less memory consuming than that of velocyto (La Manno et al., 2018).

RNA velocity inference could predict the direction of cell transition within and between cell clusters/states. By contrast, pseudotime/trajectory analysis aims to identify the paths between cell clusters/subtypes, which does not automatically infer a direction like RNA velocity prediction. However, RNA velocity analysis can benefit trajectory inference or pseudotemporal ordering that aims to deduce the order of cells along developmental paths by overlaying the directionality of velocity to trajectories to better predict cell fate decisions (La Manno et al., 2018). Therefore, integrative analysis of single-cell RNA velocity and trajectory/pseudotime could provide deeper insights into various dynamic cellular processes in development and evolution, such as lineage decisions and gene regulation.

## Inferring Chromatin Accessibility

Besides transcriptome profiling, scRNA-seq could also provide the potential for decoding the chromatin accessibility of transcribed regions in single cells (Figure 4B). Chromatin accessibility is essential for establishing and maintaining cellular identity by governing cell- or context-specific gene expression (Pennacchio et al., 2013; Klemm et al., 2019). The landscape of chromatin accessibility broadly reflects the regulatory capacity and is dynamically changing in response to developmental cues and environmental stimulation (Klemm et al., 2019). Some single-cell technologies are emerging to measure the chromatin accessibility of individual cells including single-cell ATAC-seq (Cusanovich et al., 2015), single-cell DNase-seq (Jin et al., 2015), and single-cell THSseq (Lake et al., 2018). Moreover, Yu et al. (2020) recently proposed a software, scATAC-pro, for quality estimation and visualization of single-cell chromatin accessibility sequencing data generated by different experimental protocols.

Determining the accessible genome is crucial for understanding the regulatory program of gene expression control. Many studies have demonstrated that the transcriptional activities of genes can be predicted based on the activities of associated regulatory elements (Natarajan et al., 2012; Kumar et al., 2013), but few researches investigated to what extent activities of regulatory elements can be inferred from the RNA-seq data. Gene transcription needs the chromatin to be open and accessible; thus, bidirectional interplay exists between transcriptome and regulome due to the feedback (Neph et al., 2012; Voss and Hager, 2014). Previously, Zhou et al. (2017) demonstrated that their method of BIRD (Table 1) can effectively predict the activities of genome-wide regulatory elements measured by DNase I hypersensitivity based on bulk gene expression profiles. Since scRNA-seq technologies enable capturing the gene transcriptional signals in each cell, it may be also possible to predict the regulome of cells based on single-cell transcriptomic data. Recently, Zhou et al. (2019) further suggested that the chromatin accessibility of the genome could be inferred from the scRNA-seq data of a small number of cells. But currently available methods for inferring chromatin accessibility from single-cell transcriptomic data are very few. Both experimental chromatin accessibility profiling technologies and computational methods that predict chromatin accessibility from scRNA-seq data will continue to improve. It remains an open question as to which method will be more accurate. To answer that question, a systematic and independent benchmark study in the future will be required.

Specifically, the data from single-cell RNA-seq and chromatin profiling technologies can be combined to delineate cellular heterogeneity and elucidate transcriptional regulatory mechanisms. For instance, the computational tool of SOMatic enables the integrative analysis of scATAC-seq and scRNA-seq data for gene regulatory network reconstruction (Jansen et al., 2019). ScAI can deconvolute the cellular heterogeneity based on single-cell transcriptomic and epigenomic profiles (Jin et al., 2020b). Additionally, MAESTRO supports cell clustering and automatic cell-type annotation as well as transcriptional regulator inference for both scRNA-seq and scATAC-seq datasets (Wang et al., 2020). These analyses will help us better elucidate the underlying mechanisms of gene regulation and cellular gene expression heterogeneity.

## Transcriptome Reconstruction for Novel Gene/Isoform Identification

For full-length transcript scRNA-seq data, transcriptome reconstruction at the single-cell level is promising to identify cell-type-specific genes/isoforms. Currently, the annotated genes and isoforms for many species including humans are still far from complete, and a multitude of novel protein-coding and non-coding genes/isoforms remain to be uncovered (Chen et al., 2013). One major reason accounting for this is that gene expression is often spatial and temporal specific; thus, those unannotated genes/isoforms could be only expressed in specific conditions and/or cell types/states. Since gene expression is usually heterogeneous at the single-cell level, different cell subpopulations may express unique and unannotated genes and/or isoforms that could not be identified with bulk RNA-seq data. Thus, scRNA-seq provides great potential for identifying and annotating the novel genes and isoforms.

**FIGURE 5:**
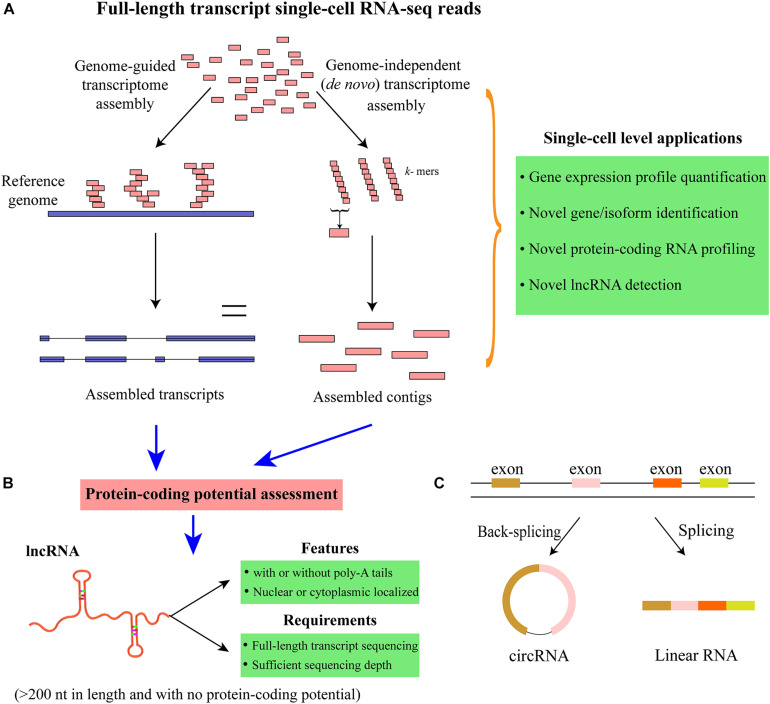
Transcriptome reconstruction and identification of lncRNAs and circRNAs. **(A)** Schematic of single-cell transcriptome reconstruction with genome-guided and genome-independent approaches. Genome-guided strategies need to map the sequencing reads to the reference genome first, whereas genome-independent (*de novo* assembly) methods can assemble the sequencing reads directly without using the reference genome. **(B)** Novel lncRNAs can be identified by assessing the protein-coding potential of the transcripts assembled from transcriptome reconstruction methods. Since lncRNAs can be with or without poly (A) tails, the full-length transcript scRNA-seq technologies that can capture both poly (A+) and poly (A–) RNAs are preferred for comprehensively profiling lncRNAs. Moreover, sufficient sequencing depth can also benefit the lncRNA identification in considering that lncRNAs are usually expressed at relatively lower levels than that of mRNAs. **(C)** Profiling circRNAs with scRNA-seq data. CircRNAs are formed by back-splicing, which is different from linear RNAs. Unlike linear RNAs that can be captured with standard poly-A enriched methods, circRNAs are covalently closed and usually need to be profiled with rRNA-depleted total RNA protocols. Furthermore, the sequencing depth is also important to ensure the accuracy of circRNA identification and quantification.

Transcriptome reconstruction is the most popular strategy for detecting all the expressed genes and isoforms in a particular sample (Garber et al., 2011; Chen et al., 2017). The approaches for transcriptome reconstruction can be mainly grouped into the following two categories: genome-guided and *de novo* (genome independent) transcriptome assembly (Garber et al., 2011) (Figure 5A and Table 1). Generally, genome-guided strategies [such as TransComb (Liu J. T. et al., 2016), StringTie (Pertea et al., 2015), and Cufflinks (Trapnell et al., 2010)] assemble the overlapping reads aligned to the reference genome into transcripts, which is suitable for the organisms with the available qualified reference genome. By contrast, *de novo* transcriptome assembly methods [e.g., Trinity (Grabherr et al., 2011), *Trans*-ABySS (Robertson et al., 2010), and rnaSPAdes (Bushmanova et al., 2019)] often utilize de Bruijn graph to directly assemble the reads into transcripts without the need of reference genome. When a qualified reference genome is available, genome-guided approaches are the choice due to their higher sensitivity than *de novo* assembly methods. However, for cancer cells, large-scale rearrangement events may exist in the genome and/or transcriptome; a combination use of these two different strategies may generate a more comprehensive set of transcripts (Garber et al., 2011). After transcriptome reconstruction, the coding potential of those assembled transcripts can be assessed to group them into protein-coding or non-coding RNAs. Although the available transcriptome reconstruction approaches are mainly designed for bulk RNA-seq data, some studies have applied them to scRNA-seq data and successfully identified many novel genes/transcripts (Yan et al., 2013; Fan et al., 2015; Liu S. J. et al., 2016; Wu et al., 2019). For example, Yan et al. (2013) integrated genome-independent and genome-guided assembly methods to predict the new transcripts and detected a set of novel long non-coding RNAs (lncRNAs) that are functionally important in human embryos. Notably, transcriptome assembly is mainly applicable to the scRNA-seq approaches that can sequence the full-length of transcripts [e.g., Smart-Seq2 (Picelli et al., 2014), SUPeR-seq (Fan et al., 2015), and RamDA-seq (Hayashi et al., 2018)] rather than the protocols that only capture the 3′/5′-end of transcripts. Moreover, novel algorithms for reconstructing single-cell transcriptome may be essential to overcome the noise and low coverage of scRNA-seq data. Overall, conducting single-cell transcriptome reconstruction is promising for identifying the novel genes and isoforms (including both protein-coding and non-coding RNAs) expressed in specific cell types/states, which may transform our understanding of the complexity of single-cell transcriptome.

## Profiling Long Non-Coding RNAs and Circular RNAs

After transcriptome reconstruction, novel lncRNAs could be identified from single cells. LncRNAs are the transcripts with >200 nucleotides in length and have no protein-coding potential. It has been shown that lncRNAs are fundamental regulators and involved in a wide range of biological processes and pathways related to transcriptional and posttranscriptional regulation as well as chromatin remodeling (Mercer et al., 2009; Slack and Chinnaiyan, 2019). Moreover, lncRNAs can play critical roles in a variety of human diseases, and some of them could be important biomarkers for many cancers (Ransohoff et al., 2018). Additionally, the expression of lncRNAs is more tissue- and cell-type specific than that of mRNAs (Ransohoff et al., 2018); thus, scRNA-seq provides unprecedented opportunities for profiling and annotating the cell-type-specific lncRNAs. To identity lncRNAs with RNA-seq data, the aforementioned transcriptome reconstruction is usually conducted to define the map of all expressed transcription units first (Figure 5B). Then a variety of methods can be applied to discriminate lncRNAs from protein-coding RNAs, such as CPAT (Wang et al., 2013), LncRNA-ID (Achawanantakun et al., 2015), and LGC (Wang G. Y. et al., 2019) (Table 1). CPAT employs a logistic regression model to discriminate between non-coding and protein-coding transcripts, while LncRNA-ID utilizes the machine learning model of random forest, and LGC is based on the feature relationship between the length of open reading frame (ORF) and GC content. The protein-coding potential assessment tools have been widely used in numerous studies to predict the protein-coding potential of transcripts, which have been reviewed previously (Han et al., 2016; Lorenzi et al., 2019).

An increasing number of studies have explored the lncRNA expression profiles and functions at the single-cell level. For example, Fan et al. (2015) developed SUPeR-seq to sequence both poly (A+) and ploy (A−) RNAs and identified hundreds of novel lncRNAs that showed developmental stage-specific expression in mouse. The random (AnchorX-T_15_N_6_) primers were used in SUPeR-seq to enable the simultaneous capture of both polyadenylated and non-polyadenylated RNAs from individual cells. Moreover, novel lncRNAs associated with human early embryonic development were identified (Yan et al., 2013), and cell-type-specific lncRNAs were observed to be abundantly expressed in human neocortex (Liu S. J. et al., 2016). Besides, Wu et al. (2019) detected over 3,000 lncRNAs using the scRNA-seq data of human bone marrow and revealed that a fraction of them could play crucial roles in dysplastic hematopoiesis. It is worth noting that lncRNAs can localize in the nucleus and cytoplasm, and are usually less abundant than mRNAs, and can be expressed simultaneously with relevant protein-coding genes. If cells can be directly lysed without RNA extraction and sequenced with substantial depth, it may allow more comprehensive lncRNA identification. Furthermore, lncRNAs can be with or without poly (A) tails; thus, the full-length transcript scRNA-seq technologies that enable total RNA [including poly (A+) and ploy (A−) RNAs] capturing [e.g., SUPeR-seq (Fan et al., 2015), MATQ-seq (Sheng et al., 2017), and RamDA-seq (Hayashi et al., 2018)] are more suitable for comprehensive lncRNA profiling, whereas those single-cell protocols that only sequence poly (A+) RNAs will miss the lncRNAs without poly-A tails. However, currently available scRNA-seq strategies that can provide whole gene body coverage are still suffering certain bias at the 3′/5′-end of transcripts; further improvement of these technologies will greatly benefit single-cell lncRNA profiling.

Additionally, circular RNAs (circRNAs) are an essential class of circularized non-coding RNAs, which are formed by back-splicing of linear pre-mRNAs (Figure 5C). CircRNAs can act as sponges for miRNAs or proteins, interfere with pre-mRNA processing, and even produce polypeptides (Lasda and Parker, 2014; Li et al., 2018). Moreover, a multitude of circRNAs have been associated with a variety of human cancers, and some of them could be important biomarkers for cancer diagnosis or prognosis (Greene et al., 2017). However, the specific functions for the great majority of circRNAs in biological systems are still unknown. Multitudinous studies have identified and annotated circRNAs with different bioinformatic pipelines based on bulk RNA-seq data (Memczak et al., 2013; Jakobi and Dieterich, 2019). However, circRNA exploration at the single-cell level is just emerging. Since circRNAs are covalently closed continuous loop and do not have poly (A) tail, they cannot be profiled with standard poly (A) enrichment protocols. Recently, an abundance of circRNAs involved in the early embryonic development of mice was identified using SUPeR-seq protocol to sequence total RNAs from individual cells (Fan et al., 2015). Furthermore, Verboom et al. (2019) proposed SMARTer technology for conducting single-cell strand-specific total RNA sequencing and detected over 500 circRNAs in neuroblastoma cell lines. A range of computational methods are available for identifying circRNAs with RNA-seq data [such as find_circ2 (Memczak et al., 2013), CircExplorer2 (Zhang et al., 2016), and CIRI2 (Gao et al., 2018)], which have been reviewed recently (Jakobi and Dieterich, 2019) (Table 1). These tools could be applicable to explore the circRNAs in single cells. The commonly used bulk sequencing strategies for circRNA detection are ribosomal RNA (rRNA)-depleted total RNA and poly (A)-depletion methods, but none of them can guarantee that the enriched RNAs are exclusively circular as some other types of ncRNAs would be also captured (Kristensen et al., 2019). By contrast, the scRNA-seq protocols for profiling circRNAs are still in the early phases of development, and the bioinformatic methods specially designed for single-cell circRNA exploration are still lacking. Furthermore, the reliable identification and quantification of circRNAs generally need a substantial sequencing depth to obtain sufficient supporting reads spanning the back-splice junction region of circRNAs.

Currently, available scRNA-seq protocols are still with high technical noise, and the sequencing depth for each cell is relatively low in consideration of the cost, which hinders the identification of lncRNA and circRNA. Additionally, the computational methods specially developed to process single-cell transcriptomic data by taking the data sparsity and noise into account for lncRNA and circRNA investigation are currently lacking. With the development of both single-cell total RNA sequencing methods and related computational approaches, exploring the lncRNAs and circRNAs in individual cells, will be more feasible. These advancements will largely promote the profiling and functional characterization of lncRNAs and circRNAs in different cell types/states under various conditions.

## Cell Composition Deconvolution of Bulk Samples Using Single-Cell Data

**FIGURE 6:**
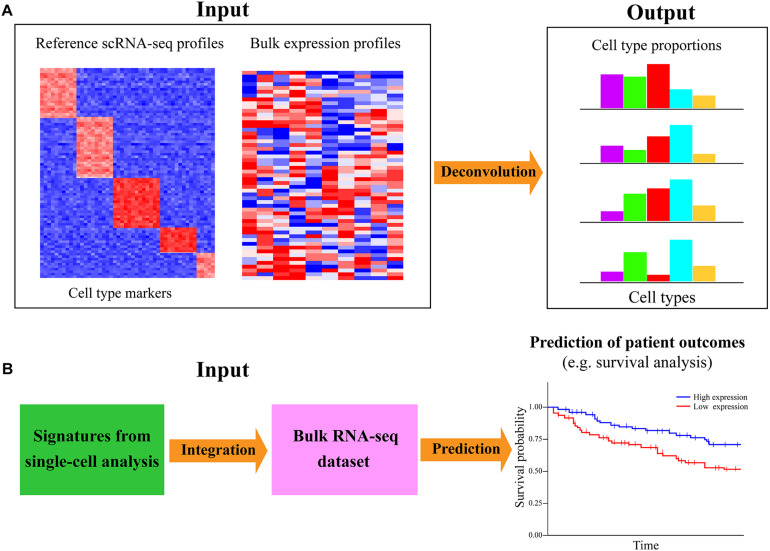
Integration of single-cell and bulk RNA-seq data for cell-type decomposition and survival analysis. **(A)** Deconvolution of cell-type composition in bulk samples with single-cell reference signatures. The computational approaches for deconvoluting the cell-type compositions or proportions of bulk samples often need the reference expression profile of the markers for specific cell types. Performing scRNA-seq on a few samples is an efficient and cost-effective way to generate the cell-type-specific gene expression profile as the reference. **(B)** Linking the expression of single-cell signatures to patient outcomes with related large-scale bulk datasets. The signatures or markers identified in diverse types of single-cell analyses (such as cell type identification, alternative splicing inference, cell–cell communication exploration, and gene regulatory network reconstruction) can be further investigated with relevant large-scale bulk RNA-seq dataset to check whether they are associated with different patient outcomes (e.g., survival of patients) based on available clinical information of involved patients. Such analysis can further gain insights into the associations of cell-type-specific markers with certain phenotypes of patients in a multitude of samples.

The aforementioned analyses are mainly based on scRNA-seq data alone; single-cell transcriptomic data can also be analyzed with the bulk RNA-seq dataset to infer the cell-type proportions/compositions for a large number of bulk samples (Figure 6A). ScRNA-seq has great advantages in dissecting the heterogeneity of cellular compositions within a given sample; however, such researches were mainly focused on a limited number of samples/individuals in consideration of cost effectiveness and scalability. Bulk RNA-seq is still the primary workhorse for dissecting gene expression for a host of samples in biomedical research due to the low cost and technical simplicity. For investigating the cell-subset specific information in a plethora of samples, an attractive approach is to directly decode the cell-type composition of large-scale heterogeneous bulk samples via deconvolution algorithms (Shen-Orr and Gaujoux, 2013). Such a strategy is not only cost effective but could also preserve both whole-system level perspective and cell-based view of cell heterogeneity. For example, Li T. et al. (2017) have explored the composition of different tumor-infiltrating immune subsets in 32 cancer types of The Cancer Genome Atlas (TCGA). Moreover, Donovan et al. (2020) deconvoluted the cellular composition of 28 distinct human tissues from Genotype-Tissue Expression (GTEx) project (Aguet et al., 2017), which allowed cell-type-specific functional investigation for the impacts of genetic variation on gene expression.

Currently, a dozen of deconvolution approaches are available for inferring the composition of cell types from bulk RNA-seq data (Cobos et al., 2018), such as CMP (Frishberg et al., 2019), MuSiC (Wang X. R. et al., 2019), DWLS (Tsoucas et al., 2019), and CIBERSORTx (Newman et al., 2019) (Table 1). CMP uses linear regression to estimate the expression abundance of reference cells in the given bulk samples, while MuSiC weights the genes exhibiting cross-subject and cross-cell consistency to transfer cell-type-specific gene expression profile across different datasets. DWLS employs a weighted least squares method to estimate cell-type proportions, and CIBERSORTx is based on the machine learning method to determine cell type abundance and cell-type-specific gene expression. A systematic comparison of the performance for recently developed deconvolution approaches is very valuable, but such a study is currently lacking. Existing deconvolution tools generally rely on the prior knowledge of reference expression profiles of known cell-type signatures, which can be obtained from the scRNA-seq data of one or a few samples (Figure 6A). At present, it is still highly expensive and time consuming to sequence a multitude of samples using scRNA-seq. Therefore, deconvoluting cell-type compositions from large-scale bulk RNA-seq dataset with a small sample size of single-cell transcriptomic data as the reference is an economically practical and time-saving way. Such analysis is valuable for identifying the cell types vulnerable to disease and detect the cellular targets of disease/cancer.

## Linking Single-Cell Signature to Patient Outcomes With Bulk Data

Another important joint analysis of scRNA-seq and bulk RNA-seq data is to associate the signatures identified in single-cell transcriptomic data exploration to predict patient outcomes. Intratumoral heterogeneity is a pivotal determinant of tumor biology, survival, and treatment response of patients. A major goal of cancer profiling studies is to identify the genetic biomarkers that are predictive for the survival status of cancer patients. The advance in scRNA-seq largely facilitates the biomarker/signature detection at a higher resolution beyond traditional bulk data. Such single-cell signatures can be screened out from different types of single-cell analyses, such as cell clustering, differential expression calling, alternative splicing exploration, and gene regulatory network inference. Specifically, important signatures could be identified from the scRNA-seq data of the tumor ecosystem to potentially predict cancer stage, therapy response, disease-free interval, metastatic probability, or overall patient survival. Although it may not be practical to perform scRNA-seq on an abundance of patients for prognosis prediction, those publicly accessible bulk datasets with available clinical information are valuable resources for such analysis. It is an alternative way to assess whether the single-cell signatures could be useful biomarkers for predicting patient outcomes (Figure 6B).

A host of studies have used the bulk datasets from public databases like TCGA (Weinstein et al., 2013) and Gene Expression Omnibus (GEO) to determine the association between the expression level of single-cell signatures and the patient survival of corresponding cancers. For example, signatures from scRNA-seq analysis were successfully applied to predict the overall survival of patients for TCGA melanoma (Nirschl et al., 2017) and hepatocellular carcinoma (Zheng et al., 2018). Furthermore, Li H. P. et al. (2017) identified single-cell biomarkers that can stratify the colorectal tumors from TCGA and GEO databases into subgroups with divergent survival. For survival analysis, Raman et al. (2019) revealed that highly variable results are usually obtained from different methods, and Cox regression (Li, 2003) is superior to other compared approaches based on tests of reliability, accuracy, and robustness. Cox regression is a flexible method that can improve the accuracy of estimation between gene expression level and patient survival by enabling the inclusion of multiple covariates to accommodate explanatory variables. It is worth noting that the single-cell signatures are used to build a model, while the actual data using the model is the bulk RNA-seq data. The continuous decreasing cost and time for scRNA-seq will make single-cell transcriptomic profiling on a large sample size become more affordable and practicable, which will greatly benefit the association analysis between single-cell signatures and patient outcomes. Consequently, the signatures/biomarkers screened out from diverse kinds of single-cell analyses could be further linked to the patient outcomes with related bulk datasets and clinical information to assess their associations and clinical value.

## Conclusion and Outlook

ScRNA-seq is widely applied to diverse organisms to dissect a range of biological questions related to developmental biology, oncology, immunology, neurology, and microbiology at the single-cell resolution. Besides those routine analyses conducted in most studies (e.g., cell type identification, alternative splicing detection, trajectory, and GRN inference), much more other valuable information can be mined from scRNA-seq data. As we summarized in this review, cell-to-cell communications, RNA velocity, and large-scale CNVs and chromatin accessibility could be effectively extracted from single-cell transcriptomic data. Nucleotide sequence changes of SNVs and RNA editing events also could be derived from scRNA-seq experiments to enable multiple modalities. Moreover, transcriptome reconstruction with full-length transcript scRNA-seq data is promising for identifying and annotating the novel genes and isoforms mainly expressed in certain cell types/states. The innovation and optimization of scRNA-seq protocols that can effectively capture both poly (A+) and ploy (A−) RNAs with increased throughput will improve the feasibility of profiling and characterizing of lncRNAs and circRNAs at single-cell resolution. Additionally, the results of scRNA-seq analysis can be further explored with traditional bulk RNA-seq data to deconvolute the cell compositions in a multitude of bulk samples or assess the association between single-cell signatures and patient outcomes in a cost-effective way.

Notably, the accuracy of any kind of single-cell analysis largely depends on the quality of single-cell sequencing data (e.g., cell quality, sequencing quality, coverage, and depth) as well as the performance of corresponding bioinformatics algorithms. Special attention needs to be paid to the noise and sparsity of scRNA-seq data, and stringent criteria may be needed to minimize the false positives. Besides, since there is a general lack of studies for benchmarking the computational approaches of the single-cell analyses we summarized in this review, it would be useful to conduct such researches in the future. In consideration of the absence of a gold-standard method, running more than one bioinformatic tools could be an effective way to reduce the number of false positives. Additionally, performance comparison for several commonly used scRNA-seq technologies revealed that if the research goal aims to pursue the highest sensitivity, the low-throughput methods that can produce full-length transcripts (e.g., Smart-seq2) are significantly better than the high-throughput approaches that mainly capture the 3′/5′-end of transcripts (like 10x Chromium) (Ziegenhain et al., 2017; Ding et al., 2020). Future comparative analysis for the newly developed single-cell transcriptome profiling protocols will be very helpful to provide better guidance in experimental designs. The fast evolution of both scRNA-seq approaches and bioinformatics methods will make the single-cell analyses we discussed become more feasible. We anticipate that these useful analyses will add much more value to scRNA-seq data and largely facilitate biomedical and clinical researches.

On the other hand, the states of single cells are determined by the intricate interplay of various molecules from multi-omic levels, such as genomics, transcriptomics, proteomics, and epigenomics. Integrative analysis of multi-omic data will enable a much more comprehensive and systematic view of each cell, which will greatly benefit the study of a variety of normal development and disease processes. An increasing number of single-cell protocols have been developed to measure different modalities including genome (Vitak et al., 2017), epigenome (Mulqueen et al., 2018), proteome (Darmanis et al., 2016), and chromatin accessibility (Cusanovich et al., 2015), as well as profile spatial (Wang et al., 2018) or lineage (Raj et al., 2018) information. Furthermore, some assays can even simultaneously capture multimodal data from the same cell (Stuart and Satija, 2019). Additionally, the third-generation sequencing technologies like nanopore can sequence RNA and DNA with super long reads (Rand et al., 2017; Garalde et al., 2018); such technological advances and improvements will effectively accelerate the refinement of single-cell multi-omic approaches. As single-cell technology matures (including sensitivity, coverage, and throughput) and the continuous decrease in cost, multi-omic studies will be more feasible and affordable. Collectively, we envision that the advances of multi-omic assays coupled with novel computational approaches will enable a more comprehensive understanding and elucidation of diverse cellular processes and significantly transform the single-cell biology.

## Author Contributions

GC conceived the manuscript. GC, YL, QX, and DW wrote the review. YL plotted all the figures. GC provided guidance on the writing and direction of the review. GC and YL revised and finalized the manuscript. All authors contributed to the article and approved the submitted version.

## Conflict of Interest

The authors declare that the research was conducted in the absence of any commercial or financial relationships that could be construed as a potential conflict of interest.
